# The power of light – From dental materials processing to diagnostics and therapeutics

**DOI:** 10.2340/biid.v11.40308

**Published:** 2024-03-18

**Authors:** Mohammed A. Hadis, Adrian C. Shortall, William M. Palin

**Affiliations:** Institute of Clinical Sciences, College of Medical and Dental Sciences, University of Birmingham, Birmingham, United Kingdom

**Keywords:** Resin based composites, light curing, high irradiance, high power, photoinitiators, dark cure, transillumination, photobiomodulation, photodynamic therapy, photodisinfection

## Abstract

Harnessing the power of light and its photonic energy is a powerful tool in biomedical applications. Its use ranges from biomaterials processing and fabrication of polymers to diagnostics and therapeutics. Dental light curable materials have evolved over several decades and now offer very fast (≤ 10 s) and reliable polymerization through depth (4–6 mm thick). This has been achieved by developments on two fronts: (1) chemistries with more efficient light absorption characteristics (camphorquinone [CQ], ~30 L mol^-1^ cm^1^ [ʎmax 470 nm]; monoacylphosphine oxides [MAPO], ~800 L mol^-1^ cm^-1^ [ʎmax 385 nm]; bisacylphosphine oxide [BAPO], ~1,000 L mol^-1^ cm^-1^ [ʎmax 385 nm]) as well mechanistically efficient and prolonged radical generation processes during and after light irradiation, and; (2) introducing light curing technologies (light emitting diodes [LEDs] and less common lasers) with higher powers (≤ 2 W), better spectral range using multiple diodes (short: 390–405 nm; intermediate: 410–450 nm; and long: 450–480 nm), and better spatial power distribution (i.e. homogenous irradiance). However, adequate cure of materials falls short for several reasons, including improper selection of materials and lights, limitations in the chemistry of the materials, and limitations in delivering light through depth. Photonic energy has further applications in dentistry which include transillumination for diagnostics, and therapeutic applications that include photodynamic therapy, photobiomodulation, and photodisinfection. Light interactions with materials and biological tissues are complex and it is important to understand the advantages and limitations of these interactions for successful treatment outcomes. This article highlights the advent of photonic technologies in dentistry, its applications, the advantages and limitations, and possible future developments.

## Introduction

The development of materials science, polymer- and photochemistry, photophysics, optical engineering, and a greater understanding of photobiology has allowed a variety of industries to harness the power of light for a range of applications from therapeutics to materials processing. This includes the field of dentistry which has embraced advancements in new light curable materials and light-based technologies to enhance patient care and treatment outcomes. Most notably, the use of lights for photocuring of polymeric dental materials has been the state of the art since the introduction of visible light curing in the 1970s. Broadly, similar principles and chemistry used for dental curing now also apply in modern additive manufacturing techniques which utilise stereolithographic or digital light projection technologies. The latter requires photoinhibitors and optimisation of radical inhibitors, photoinitiator chemistry and viscosity to control spatial and temporal resolution during curing in order to incrementally cure thin (microscale, <100 µm) layers of photopolymers for the fabrication of 3D structures and objects [1–3]. Indeed, additive manufacturing and digital technologies have also rapidly gained interest in dentistry in recent years owing to advancements in these areas, their low cost, and efficiency in production [3].

In comparison to industrial photopolymer applications, such as photoresists and coatings which utilise ultraviolet (UV, 300–400 nm) free radical photoinitiators that provide superficial surface curing in the micrometre scale, modern photocurable dental materials use visible violet and blue light photoinitiators (typically 400–500 nm). The choice of photoinitiator system and light source is critical in many aspects including curing efficiency, depth of cure, mechanical properties, and biological safety. The use of visible light photoinitiators mitigates risks associated with higher energy of UV photons on biological tissues and also overcomes the limitations associated with poor transmission of UV light in highly scattering materials [4–7]. Modern dental lights include lasers and LEDs of various sizes, wavelengths and design, and are now also emerging as technologies to revolutionise other aspects of dental practice, from diagnostics to restorative and cosmetic/aesthetic procedures, and therapeutic applications which include photobiomodulation (PBM), photodynamic therapy (PDT), and photodisinfection. The use of photonic energy to cure materials, illuminate, visualise and inspect oral tissues, as well as to manipulate and treat oral tissues and control infections is a rapidly growing area of dentistry. By harnessing the power of light, dental professionals can achieve greater precision, efficiency and patient comfort, ultimately improving the quality of dental care. This article aims to explore these diverse and emerging applications of lights in dentistry, highlighting their contribution, benefits, limitations, and exploring how they are a key element of modern dental practice.

## The evolution of light curing

In the last 5 years, there has been a reported year on year increase in the number of publications relating to photocurable dental polymers and commercially marketed products. Much of the research and developments has focussed on enhancing mechanical, physical, and biological aspects with 37.6% of studies being related to the silica glass filler material which includes bioactive and fibrous types [8]. However, at the core of light-based technology in dentistry is the dental light curing unit (LCU), an essential item of equipment in contemporary dental practice, specifically designed for intraoral use to crosslink photopolymer restorative materials in situ. Light energy (photons) activates a photoinitiator within the material, triggering a cascade of photochemical reactions that leads to the rapid polymerisation and hardening of the material. The ability to harness light energy for crosslinking polymer materials has significant advantages in terms of spatial and temporal control of the setting reaction to allow macroscale incremental build-up of restorations (typically less than 6 mm thick) [9, 10]. Inadequate light curing may jeopardise the success of many dental procedures [11]. Appropriate radiant exposure (the total energy delivered per unit area [J/cm^2^] given by irradiance [W/cm^2^] × exposure time [s]) is required to adequately cure any photocurable polymer-based composite restoration. For these reasons, both the photoinitiator system [12–25] and the dental LCU [6, 26–36] have also been subject to much research and development, particularly with the introduction of new shorter wavelength, more efficient and faster reacting photoinitiators over the last two decades. The diversity in photoinitiator chemistry and light curing technologies has also led to guidelines for proper selection, maintenance and use of LCUs to be published [37–40]. These trends are likely to continue with a substantial growth in the dental light curing markets predicted in the next 10 years [41, 42]. The latest developments include materials optimised for rapid polymerisation with so-called ‘high power’ (typically ≥1 W) LCUs that deliver high irradiances (>3,000 mW/cm^2^) in short exposure times (≤ 3 s) [24, 43–46]. Such developments are not only achieved through LED and optics technology, but also optimisation of materials chemistries including the type, composition and concentrations of polymers and fillers, and the type and concentration of pigments, dyes and photoinitiators, which are aimed at optimising light transmission and photon delivery [9]. However, while rapid polymerisation offers time saving advantages, one of the major associated limitations is the increased shrinkage stress compared with low irradiance curing protocols which has been a major focus of composite development over the last two decades [43]. Evidently, the light curing procedure and the type of photoinitiator is one of the most important components to optimise mechanical, physical, biological and aesthetic properties of dental materials. While guidelines for the selection, use, and maintenance of LCUs are available [39, 47, 48], clinical variables influence treatment outcomes. Achieving and maintaining correct stable light guide positioning throughout cure becomes more difficult in posterior locations and operator variability is a critical factor in regard to energy delivery [49, 50]. Allied to this fact, many dentists do not look into the patient’s mouth during the light curing procedure [51]. Education and proper training have proved to be effective for improving light curing skills [51]. Indeed, clinical failure rates of photocurable restorations have been linked with improper placement and curing of materials, currently ranging from 0.08 to 6.3% [52]. Thus, a fundamental understanding of the materials photochemistry and photophysics is important, and it is timely to review the state of the art for photocuring of dental materials in terms of the photochemistry and photophysics involved.

### Photocuring

The use of alternative photoinitiator systems or a combination of photoinitiator systems in dental materials has steadily increased since the first inception of visible light curing [12, 15, 18, 20, 21–24]. Such systems provide unique advantages in terms of curing efficiency, mechanical properties, and biological safety. However, multiple initiators or even single photoinitiators with different absorption profiles and absorptivity compared with commonly used camphorquinone (CQ), require specifically engineered dental lights. These are often multiple-diode LED curing lights that provide specific initiation wavelengths required for each photoinitiator in order to achieve faster polymerization rates and to provide appreciable initiation rates at depth [25, 53]. Unfortunately, most manufacturers are reluctant to divulge the chemistry of their photoinitiator systems which they consider proprietary, making it difficult for proper material-curing light selection beyond specific lights that are recommended by the materials manufacturer. While it is good practice to follow the recommendations of the manufacturer, this may not always happen due to several reasons which include a poor understanding of the materials chemistry, properties of lights available in dental clinics or even the prohibitive expense of purchasing new devices. Therefore, the purpose of this section is to provide an overview of the photoinitiators and the associated photochemistry in dental materials.

### UV initiation

The first light curable dental polymer-based composite was developed around 1970 for class III-V restorations by Buonocore and Davilla in 1973, utilising a Norrish Type I UV initiator, benzoin methyl ether which allowed a 50-fold increase in light absorption compared to benzoin. The mechanism involved direct absorption of light at ~365 nm to generate free radicals through homolytic scission of the central bond which is weakened by the electron withdrawing tendency of the two oxygen atoms present. The short wavelength absorption required a relatively expensive high-pressure mercury arc light source, Nuva Lite (L.D. Caulk), that emitted wavelengths between 340 and 380 nm, directed through a quartz rod. The relatively low irradiance (10–50 mW/cm^2^) by two orders of magnitude compared with modern LED LCUs (>2,000 mW/cm^2^) and ISO standards (minimum 300 mW/cm^2^) resulted in recommended exposures of 60 s, with limited depth of cure, around 1.5 mm (or 750 µm by modern ISO standards [54]). Such limited depth of cure was likely due to a combination of several factors including the relatively low curing irradiance [9], the high molar absorptivity typically found in the Type I photoinitiators which limit light transmission through depth [12], and the absorption and scattering characteristics of tooth tissue and the composite materials particularly with the short wavelengths of light that was needed [55–57], making the restoration accessible for exposure effectively only from its free surface. Since the curing proceeded essentially from the exposed surface down, it was difficult to cure restorations in depth without utilising long exposure times [9]. In fact, even longer exposure times were required to improve energy delivered to the surface and through depth owing to the steadily declining efficiency of the light source with age which compensated for the reduced output irradiance, but this compensation was based mainly on guesswork and therefore resulted in unpredictable and unreliable curing. In addition, the use of short wavelengths in close proximity to soft tissues poses serious health and ocular risks to the patient and operator (including ‘sunburnt’ gums and increased risks of developing cancerous lesions if high energy UV light used), with risks amplified with decreasing wavelength and increasing exposure time, therefore requiring considerable care generally [7, 58]. Despite the limitations of UV curing, a 17-year long clinical follow up of four commercially available UV cured posterior class I and II composite materials found that 76% of the recalled restorations were clinically acceptable [59]. This extraordinary result when compared to results from most clinical trials of contemporary materials is likely due to good operative practice and patient selection. Nonetheless, the use of UV-curable dental polymer-based composites were short lived and rapidly replaced with visible light photoinitiators which absorbed longer and much safer visible wavelengths providing an alternative route to the generation of free radicals. This remains state of the art in most modern polymer-based restorative materials today.

### Visible light curing

**Figure 1:**
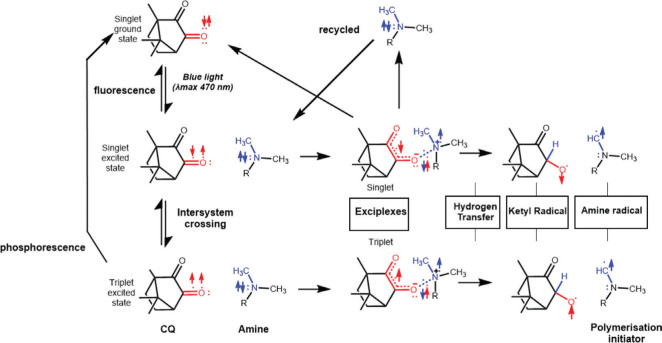
The generation of polymerisation initiators in CQ-Amine systems. Electrons are excited to a singlet state with anti-parallel electron spin which can easily return to ground state through fluorescence (emission of light at longer wavelength). Intersystem crossing to the triplet state results in a parallel spin pair where return to ground state is forbidden by quantum rules and therefore the triplet state is long lived. A electron spin flip in the triplet state may result in a return to ground state through phosphorescence. Radicals generated through the triplet state are the effective polymerisation initiators since recombination of parallel electron spins is forbidden by quantum rules. Adapted from [60].

From the first inception of visible light curing in dentistry (~1970s) to most modern photocurable dental materials, the diketone CQ with a tertiary amine has been used as the ‘gold standard’ polymerisation initiating system with the mechanism often referred to as Norrish Type II photosensitized reaction. Indeed, the photosensitisation mechanism of CQ is similar to a Norrish Type II photoreaction but the intermediary steps leading to radical generation are somewhat different and does not involve homolytic bond cleavage as would be expected with Norrish Type II photoinitiators [60]. In both cases, free radicals from visible light curing are produced through the absorption of light by a photosensitiser which in its ground state is capable of absorbing radiation resulting in the excitation of a non-bonding electron to an excited state known as a singlet state, while preserving electron spin direction. This singlet state is short lived due to the preservation of the spin direction resulting in rapid decay (~10 ns) and return to ground state with the emission of radiation at longer wavelength and lower energy through a fluorescence process which thermalizes some of the absorbed energy [61]. However, there are many electronic energy states available for electrons, including a triplet state that is similar in energy to the singlet state but can be occupied by electrons in parallel spin to the singlet state. Occasionally, through molecular collisions, an electron can flip its spin and occupy the triplet state through another process known as intersystem crossing without breaking quantum rules since both states are of similar energy. A return to ground state requires a simultaneous change in electron spin and energy levels which is forbidden by quantum rules, thus resulting in a population inversion to the high energy, long lived -triplet state (Figure 1) [61]. This provides sufficient time for this energy to be transferred to a second molecule to generate the radicals required for photoinitiation, either through homolytic bond cleavage (Norrish Type II mechanism) or a photosensitised redox mechanism (CQ) [60]. For the latter, the excited state CQ reacts with the tertiary amine to form an excited state complex, known as an exciplex. The formation of the exciplex provides an intermediatory step that allows internal hydrogen transfer from the amine to the CQ and conversion to a pair of free radicals. The radicals that are generated are of two types; (1) a terminating ketyl radical which is not involved in polymerisation, and (2) amine radical which initiates polymerisation. Thus, this polymerisation initiation system is a redox system because the transfer of an electron between molecules is involved in the crucial step. The amine is therefore an electron donor or reducing agent and the CQ is an electron acceptor or oxidising agent and neither are considered to be true ‘photoinitiators’.

**Figure 2:**
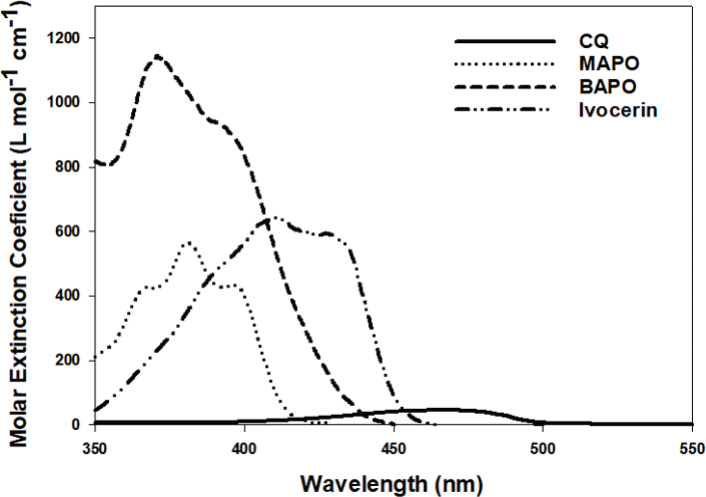
The molar extinction coefficient of four common dental photoinitiators measured in methyl methacrylate. Type I photoinitiators typically absorb at shorter wavelengths (<445 nm) usually with much greater absorptivity (as much as ~20× greater than CQ, ~30 L mol^-1^ cm^1^ (ʎmax 470 nm); e.g. monoacylphosphine oxides (MAPO), ~550 L mol^-1^ cm^-1^ (ʎmax 385 nm), bisacylphosphine oxide (BAPO), ~900 L mol^-1^ cm^-1^ (ʎmax 385 nm), Ivocerin, ~600 L mol^-1^ cm^-1^ (ʎmax 408 nm). (unpublished data).

**Figure 3:**
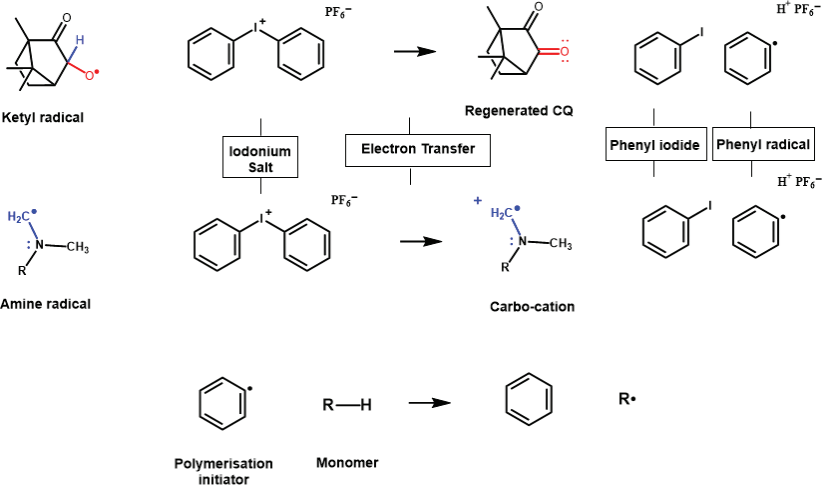
The photoinitiation mechanism of CQ/Amine in the presence of iodonium salts. The intermediate steps for the generation of the ketyl and amine radicals are shown in Figure 1. The presence of the iodonium salt improves efficiency of the CQ-Amine reaction through its strong oxidising potential. Firstly, electron transfer from the ketyl radical regenerates CQ and also generate an additional polymerisation initiating phenyl radical. Electron transfer from CQ and amine to iodonium salt also prevents back-electron transfer processes thereby further improving CQ-Amine efficiency.

The use of photosensitiser such as CQ lowers the energy requirement for excitation of electrons to the singlet state compared with UV initiators so that the corresponding radiation required for absorption is a longer wavelength, and lower energy visible blue light (ʎmax ~468–470 nm depending on the solvent it is measured it) and thus provides an alternative to the use of UV light. There are several key advantages which address the limitations of UV curable materials, most notably reduced risks associated with high energy UV light [4, 60], the ability to utilize cheaper tungsten filaments (quartz-halogen), although this type of light source is no longer manufactured for dental light curing applications, or modern LED technology [37, 38], and reduced absorption and scattering of longer wavelengths of light by tooth tissues and highly filled dental materials [55–57]. In addition, CQ strikes a good balance of molar absorptivity level for deep curing, and one reason why it remains state-of-the-art for commercial dental polymer-based composites [9, 12]. However, there are several limitations which relate to both the photosensitiser and the tertiary amine. Firstly, the partly unbleachable chromophore of the diketone compromises the ability to colour match in light or bleach shades [13, 14, 26]. Secondly, the bimolecular reaction between CQ and amine results in much slower polymerisation reaction kinetics compared to Type I photoinitiators [9, 12, 15]. This is partly due to the much lower molar absorptivity of CQ compared with Type I initiators (Figure 2) and partly due to the presence of terminating ketyl radicals and processes such ‘back-electron transfer’ [12, 15, 62]. Finally, the amine itself can be problematic, with increased risks of cell mutagenicity and reduced colour stability [16–17]. Several strategies exist, which are aimed at overcoming these limitations including a reduction in the concentration of amines [16–17, 62, 63], the use of more bio-friendly amine free co-initiators [16], or the use of alternative photoinitiator systems [12, 15, 18, 20]. Each of these strategies has a significant impact on polymerisation efficiency, degree of conversion, mechanical properties, cosmetics, and biocompatibility. However, it is likely that the main focus of most commercial developments has been to improve material properties and reaction efficiency, likely due to the greater demand and commercial potential for faster and more efficient curing [9, 12, 15]. Indeed, the use of multiple component photoinitiator systems such as ternary systems that incorporate iodonium salts or additional Type I photoinitiators are now common place in modern photocurable materials [9, 18–21]. For the former, the iodonium salt, diphenyliodonium hexafluorphosphate has been used in commercial dental polymer-based materials as a third photoinitiator component to essentially improve the efficiency of the CQ-amine reaction [23]. The mechanism involves an irreversible electron transfer from the ketyl or amine radical to the iodonium salt (Figure 3). The iodonium salt is then able to rapidly fragment into a molecule of phenyl iodide and a phenyl radical. These irreversible reactions generate the original CQ molecule and also convert the amine radical into a carbo-cation. Thus, the increased efficiency of the three-component CQ-amine-iodonium salt system is mainly due to two main factors. Firstly, the terminating ketyl radical is consumed through oxidation by the iodonium salt to yield an additional phenyl radical, which, unlike the original ketyl radical, is active for initiation. Secondly, in the absence of the iodonium salt, the electron transfer from the amine to the CQ is reversible leading to ‘back-electron transfer’ which is prevented in the presence of iodonium salts. This results from the ketyl radical being reduced by the iodonium salt thus preventing the back electron transfer process [62]. Back electron transfer reduces the efficiency of the photopolymerisation process and results in inefficient curing. By inhibiting this process, and by generating another active radical, a higher concentrations of radicals is produced and maintained for an extended period. This allows for a more efficient polymerisation leading to improved mechanical properties and hopefully also clinical outcomes [21–23, 62].

**Figure 4:**
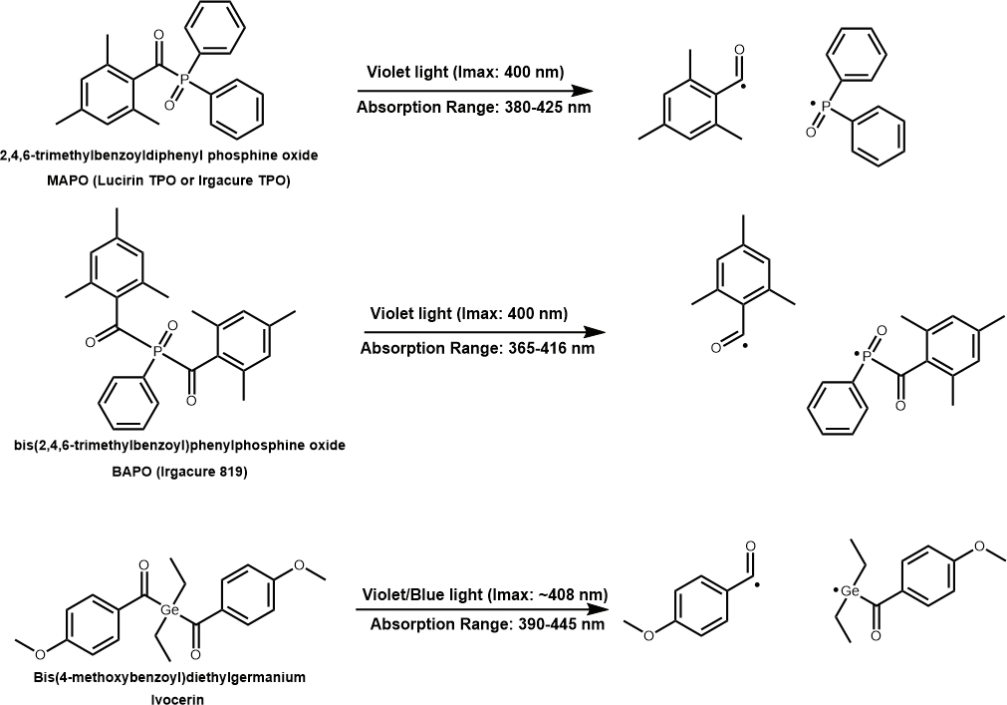
Type I photoinitiators used in dental materials and the polymerisation initiating radicals generated through homolytic scission. The efficiency of the initiators depends on the reactivity, the molar extinction coefficient, and the absorption wavelength range. Each initiator generates two active polymerisation initiating radicals with the phenyl phosphine radical being more reactive in bisacylphosphine oxide compared to monoacylphosphine oxides.

Other manufacturers incorporate Type I photoinitiators which absorb at shorter wavelengths ~385 -445 nm (although the use of lights below 400 nm is not approved for dental use by the Food and Drug Administration due to safety risks associated UV radiation) usually with much greater absorptivity (as much as ~20 × greater than CQ, ~30 L mol^-1^ cm^1^ (ʎmax 470 nm); for example monoacylphosphine oxides (MAPO), ~550 L mol^-1^ cm^-1^ (ʎmax 385 nm), bisacylphosphine oxide (BAPO), ~900 L mol^-1^ cm^-1^ (ʎmax 385 nm) [12, 15]) (Figure 2). The mechanism of these Type I initiators involves the direct absorption of light leading to homolytic scission of the molecule to more efficiently generate polymerisation initiating radicals (Figure 4). This strategy has two primarily aims, firstly to reduce the concentration of CQ and hence its yellowing effect for aesthetic purposes, and secondly to improve curing characteristics through depth [9]. The premise of the latter is to provide both advantages of a higher (more efficient and faster reaction) and lower molar absorptivity (safer visible light and improved light transmission through depth), irradiated using different initiation wavelengths appropriate for the absorption properties of both initiators. Indeed, previous work has explored the sole use of MAPO as a one-component photoinitiator system in filled experimental materials cured for 1 s at 1,000 mW/cm^2^ with significantly increased polymer conversion and minimal monomer elution (following 1 week immersion in a 75% ethanol solution) compared with CQ-based materials irradiated for 20 s [24], albeit with significantly reduced curing depths in the former (2.5–3.9 mm compared with 7.3–10.8 mm, respectively) [15] as a result of the higher molar absorptivity.

A further example of modern photoinitiator developments in commercial dental products includes the use of benzoyl germanium derivatives. Specifically, the use of benzoyltrimethylgermane and dibenzoyldiethylgermane, amine-free, one-component (Type I) initiator systems were reported with higher molar absorptivity ~700 L mol^-1^ cm^-1^ (ʎmax 408 nm) [64], higher photo-reactivity, increased quantum yield (0.85 compared with 0.55–0.59 for acylphospine oxides [15,18] and <0.10 mol einstein^-1^ for CQ-based systems [19]) and the potential to reduce curing time and increase curing depth [20]. Although higher in price, there are also numerous other advantages which include low toxicity and greater thermal stability compared with other Type 1 photoinitiators [64]. Synthesis of an optimal derivative, bis-(4-methoxybenzoyl)diethylgermane was patented in 2009 under the commercial name, Ivocerin™ (Ivoclar-Vivadent, Liechtenstein) [65]. Other germanium-based compounds such as triphenylgermanium have also been explored for use with CQ and hydride/iodonium salts as amine free initiating systems with reportedly excellent degree of conversion and photobleaching properties [66].

### Dark cure

The use of alternative photoinitiators and a combination of photoinitiators is well explored [12–25] and although the degree of polymer conversion for adequate clinical performance has yet to be established, most modern materials cure between 50 and 75% carbon double bond conversion irrespective of photoinitiator type when cured using sufficient light irradiance and exposure times [67, 68]. This includes conventional materials cured at 2 mm thick increments [68] as well as modern bulk materials which have supposedly optimised light transmission through depth with comparable polymer conversion reported for depths of up to 4 mm [69, 70]. However, it has been shown that the depth of cure is product dependent and although adequate polymerisation can be achieved at depth, the bottom is usually less well cured in bulk fill materials compared with conventional materials which is likely due to the lower light irradiances delivered in deeper increments [71]. Although the cure is likely to improve during the post-cure phase [72], conversion plateaus rapidly after the light is switched off as radical production ceases while termination continues [73, 74]. Due to this plateau, it has also been reported that the post-cure of some bulk fill materials may exceed the generally accepted 24 h to reach adequate conversion compared with conventional materials [75]. Thus, it is apparent that the ‘dark-cure’ reactions of light curable materials are as equally as important as the initial ‘command’ set of the material by light, which is an area of interest in modern developments.

**Figure 5:**
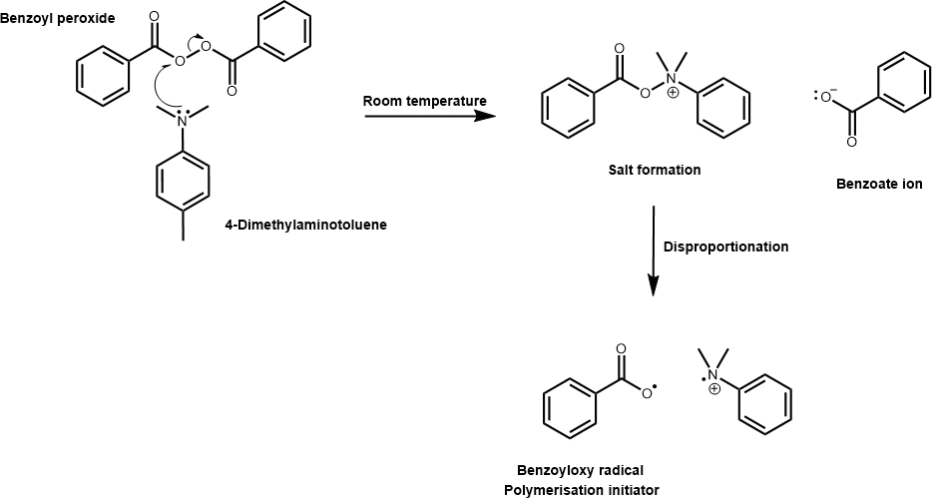
The activation of Benzoyl peroxide by a REDOX (reduction-oxidation) mechanism using a reductant. For self-cure and dual cure materials, the reductant is usually a tertiary amine such as dimethylaminotoluene and is supplied separately to the oxidant (Benzoyl peroxide). Mixing results in the formation of a salt which then disproportionates to generate the Benzoyloxy radical which can initiate the polymerisation reaction.

Indeed, the increasing acceptance of new ‘fast-curing’ photoinitiator chemistries is still overshadowed by the uncertainty of an adequate curing depth [76]. Chemically cured (self-curing polymerisation) materials overcome the limitations of inadequate light transmission altogether and offer advantages in terms of the ability to fabricate very thick materials with perhaps unlimited cure depths, although this is at the cost of losing temporal control of the reaction since the base (organic peroxide) and the catalyst components (organic amine) require mixing (Figure 5). Such materials have failed to gain popularity for several reasons. Firstly, there is a potential for non-homogenous mixing since the two components need to be folded either by hand or Kenics mixers, which can lead to differential reaction exotherms in the material and differential cure [60]. Secondly, since the components are folded in a non-vacuum environment, the inclusion of air bubbles is inevitable which act as porosities that can weaken the overall mechanical strength [60]. The mechanical strength is also further reduced by the requirements of low viscosity components to enable mixing through a Kenics syringe, which is often achieved by reducing the filler load or increasing the amount of low molecular weight monomers [46]. In addition, unlike light-cured materials which allow curing on demand, the redox activation process of chemically cured materials occur on a different time scale (minutes or hours rather than seconds) and although this may be advantageous for stress relaxation [77], it may not meet modern clinical expectations for fast restorative procedures. So called ‘dual-cure’ materials are designed to leverage the benefits of both chemically cured and light cured materials and claim to offer a balance between temporal control, cure depths, speed, and adaptability in various applications but so far have mainly been used for core build-up and cementation only [78]. In these materials, both initiation systems work independently and the same limitation in terms of light transmission, mixing problems and setting times remain. For these reasons several concerns have been raised, including whether the self-curing polymerisation reaction is sufficient in low irradiance conditions or in the absence of light altogether. Indeed, a hardness reduction in the middle of the bulk for some dual cure materials has been reported compared with the hardness of the irradiated surface or at greater depths [79]. This firstly suggests that like light ‘only’ cured materials, light transmission is a limiting factor in ‘dual-cure’ materials. Secondly, the authors of that article also suggested that the lower irradiances delivered in the centre compared to the high irradiance on the top surface and the zero irradiance on the bottom surface, interfered with the chemical-curing mechanism in the middle region [79]. On the other hand, it has also been reported that some materials behave similarly in terms of hardness and elastic modulus at observation times above 11 min after mixing regardless of curing procedure [80, 81] which further highlights the variability between materials in how they cure. The same authors also showed accelerated polymerisation kinetics at the initial stages of polymerisation with additional curing but without any detrimental effects on the final properties [81]. More recently, the crucial role of light curing for adequate polymerisation of ‘dual-cure’ materials was reported by Windle et al. [79] in which they found higher surface hardness for ‘dual-cure’ materials that received photoactivation compared to without photoactivation with depth of cure being product dependent. More importantly, they also found that the extent of the self-cure in three out of four ‘dual-cure’ materials tested was influenced by the amount of light they received with only one material which showed continuous cure up to 6 mm regardless of self-cure or light cure mode. Interestingly however, the authors also reported detrimental effects of using low irradiance in dual cure materials with better cure being reported beyond the surface without light curing. Another similar study highlighted a statistically significant correlation between radiant exposure and microhardness in which the authors reported no detrimental effects of high irradiance curing on polymerisation except in one material that was sensitive to the combination of irradiance and exposure time used [82]. In that material, high irradiance curing resulted in 20% reduction in micromechanical properties which is most likely due to higher free radical termination rates and the formation of short polymer chains on the surface [83].

New initiator chemistries have thus recently been developed which allow photocuring of very thick materials with very low irradiances although with long exposure times [84]. In that study, unfilled specimens were cured to depths of 8.5 cm using 110 mW/cm^2^ for 20 min. This was achieved through the generation of charge transfer complexes (CTCs) between an iodonium salt, phosphine additives and an amine electron donor which all had very low molar absorptivity at 405 nm thus allowing improved light transmission through depth which extended the depth of polymerisation. While this was promising in terms of depth of cure, polymer heterogeneity and non-homogenous conversion profiles that ranged from ~70% (top) to ~50% (at 8 cm depth) were identified as potential drawbacks. Furthermore, the high optical transparency and the very long exposure times meant these materials were not suitable for dental applications.

**Table:** 

Light	*Peak Wavelength (nm)	**Power (mW)	*Irradiance (mW/cm^2^)
**Halogen**	480	290	1249
**Plasma light**	476	814	1037
**Single Peak LED**	453	743	2120
**^§^ Polywave LED**	456	357-1207	**High**:1220, **Power**: 3137, **Pre**: 930, **Tack**: 2139
**Laser Diode**	457	~1800	1539

**Figure 7.** *Wavelength and power determined using a spectrometer-based device (MARC LC, Blue light Analytics except

§ Polywave LED which was measured on a STS Spectrometer, Ocean optics UK)

** Power is determined using power meter/PD300 or fast response meter

**Figure 6:**
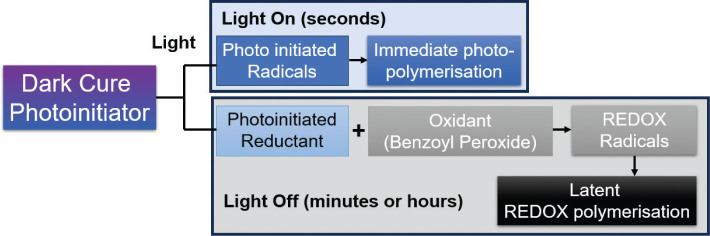
Schematic of novel ‘dark-cure’ photoinitiators which are activated by light to generate photo-radicals and a photo-base (reductant). Since the generation of the photo-reductant is activated by light, these materials are stable as one component systems (unlike dual-cure materials). The generation of photo-radicals leads to immediate photopolymerisation (in seconds) whereas the generation of the photo-base leads to latent REDOX (reduction-oxidation) polymerisation through reaction with an oxidant (e.g. Benzoyl peroxide over minutes and hours rather than seconds). This latent REDOX polymerisation supposedly increases the degree of conversion in the absence of light (dark-cure).

Stansbury’s group have also developed a quasi-biomimetic approach which involves the use of a photosensitiser (methylene blue, absorption in the visible wavelength range), an amine reducing agent, and an oxidising iodonium salt. The photoinitiated polymerisation involves organic photoredox catalysis reactions that restore the metastable reactants to sustain radical formation hours after initial and relatively short, low irradiance exposures [85]. The authors reported the ability to achieve ~80% carbon double bond conversion without significant variation through depth in 1.2 cm thick HEMA samples after ~120 min following exposure of only 3.4 mW/cm^2^ for 60 s in which the initial carbon double bond conversion during light irradiation was a mere ~8%. Under similar conditions, conventional CQ/amine photoinitiator systems exhibited a steep reduction in conversion to zero even though the optical transmission was 10 times greater compared with the organic photoredox system [85]. While this was promising, the use of methylene blue as a metastable reactant that can be restored after irradiation meant unavoidable and unacceptable blue colouring in the final polymer due to absorption at ~650 nm and therefore could not be practically considered for dental applications. The same group later reported the development of so called ‘dark-cure photoinitiators’ (DCPI’s) without distinctive colouration as a result of the UV absorbing benzophenone chromophore used as the DCPI scaffold (Figure 6) [73]. Under light irradiation these DCPI’s generate photo-radicals which initiate polymerisation, and a photo-base that releases amine reductants that undergo ground-state redox reactions with a peroxide for latent generation of radicals over extended periods which allow prolonged post-irradiation conversion. Since both mechanisms are activated by light, unlike ‘dual-cure’ materials, these DCPI’s are therefore stable as one component formulations prior to irradiation, thus overcome the limitations of mixing [73]. While the initial results were again promising with ~23% additional conversion (dark-cure) after initial irradiation using 30 mW/cm^2^ to 20% photopolymerisation (< ~3 min), absorption by the benzophenone chromophore within the UV range meant short wavelength UV light (365 nm) was required which again was not suitable for dental applications.

More recently, through rational design and development the same group reported and patented new visible light DCPIs based on quaternary ammonium salts, tertiary amine cations and a borate anion constituent [86]. In particular, 5,7-dimethoxy-6-bromo-3-aroylcoumarin-DMPT/BPh4 has been shown to facilitate ‘dark-curing’ mechanisms by concurrent photo-radical generation and photo-induced release of an efficient redox reductant under visible irradiation using 3-aroylcoumarin as the chromophore scaffold. The reductant-tethered chromophore showed strong molar absorptivity at 405 nm (5,710 M^-1^ cm^-1^) which is more suitable for dental applications than previously developed UV absorbing DCPIs [73] although weaker molar absorptivity (50 M^-1^ cm^-1^) at 455 nm was reported. The authors further reported significant photo-bleaching which reduced the internal filter effect which not only increased the depth of cure prior to the redox component of the mechanistic cascade but also the speed of the light curing to 20% conversion. Importantly, the results showed >35% additional conversion in methacrylate polymers over 25 min (although it is likely to further increase with time) following the initial irradiation to 20% conversion and the ability to rescue under-cured regions post-irradiation [74]. However, whether this can be achieved in all methacrylate based dental polymers is yet to be demonstrated.

### Curing light development

**Figure 7:**
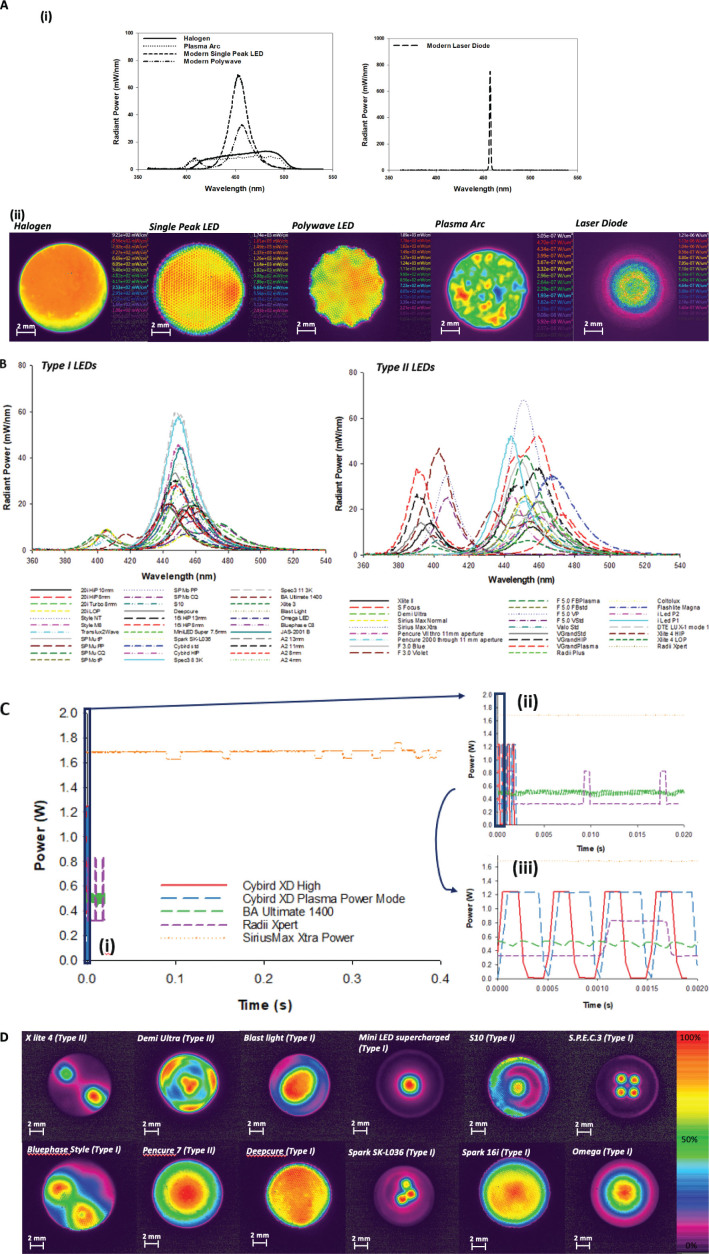
(A) Examples of different type of dental light curing unit (LCU) technology and their radiometric properties. Fig a(i) shows examples of the spectral irradiance of different light source type. The details for manufacturers of the lights and spectroradiometric information is displayed in the accompanying table. Fig a(ii) shows beam profile of the same example halogen, modern single peak LED, modern polywave LED, Plasma Arc light and Laser Diode. (unpublished data). (B) The diversity in spectral radiant power for examples of modern LED light curing units. ‘Type I’ refers to LED LCUs with graded fiber bundle light guides and ‘Type II’ refers to LCUs with the light source in the head of the unit. A broad range of spectral power can be seen with LED LCUs exhibiting single peak outputs or multiple peak outputs, typically between 380 and 500 nm. Figures adapted from Shortall et al. 2021 [27]. (C) A diversity in beam modulation can be seen for several examples of LED light curing units (LCUs) in different modes of operation. Figures 7C (i-iii) but on different timescales to show differences in pulse frequencies and amplitude. Figures adapted from Shortall et al. 2021 [27]. (D) Examples of beam profiles in dental LCUs. The images represent how the total optical power of the LCU is distributed over the optically active area from highest (red) to lowest (purple). It can be seen that the variation in beam profile is a result of factors including light guide type, the number of diodes present, diode wavelengths and the optics used. The light output scale here has been converted to percentage of maximum irradiance output from each individual LCU to allow easier comparison of beam profile. (unpublished data).

Since the development of light-activated polymer-based composites in the 1970s, more than 1,000 dental LCUs brands have been marketed, leading to diversity in spectral irradiance, optical power, peak wavelength, pulse modulation, beam profiles and energy delivery (Figure 7 and Table 1) [27]. Quartz tungsten halogen (QTH) LCUs, for over 30 years and up to the new millennium, were the mainstay of LCU technology from the mid-1970 to the mid-2000s but have been progressively replaced over the last two decades with advancement in new LED technologies [27, 37, 38]. Other technologies such as high-powered plasma-arc devices, zinc and sodium high pressure lamps [87], indium high pressure lamps [88] and Argon lasers [89], introduced in the 90s have failed to transition into suitable replacements for QTH-LCUs due to high costs, their ineffective curing peaks and complexity of use [28]. Indeed, superior results have been reported using pulsed lasers as opposed to conventional methods at the time, but this was attributed to several reasons including the high energy 10 mJ pulses and 20 ns duration at 10 Hz, and the monochromatic 468 nm wavelength of light which perfectly coincided with the absorption maxima of CQ [90, 91]. A subsequent follow up paper also found that the pulsed laser gave equal cure in depth for one fifth of the energy of a QTH unit [92]. It is likely that this technology was never adopted due to the high cost of lasers and laser modulation at the time and therefore this work has never been confirmed. The success of LED-LCUs have therefore been attributed to several reasons which include their lower costs, improved energy efficiency which allows reduced power consumption and therefore more convenient battery powered operation, higher and more stable power output that leads to more reliable curing [27, 37, 38] which also supposedly allows a reduction in curing time (from ~40 s for QTH, <0.5 W to ≤3 s for high powered LEDs, <1 W), claims of longer service life, and its more ergonomic and compact design [39, 93].

**Table 1:** Examples of Type I (above) and Type II (below) light curing unit optical properties and tip dimensions.

Light curing unit	Manufacturer/ Distributor	External tip diameter* (mm)	Active diameter** (mm)	Wavelength Range (nm)	Violet/ Blue Chip	Peak Irradiance* (mW/cm2)
**Bluephase 20i**	Ivoclar	8 10	7.4 9.0	385-515	V/B	650, 1200 & 2000
**Bluephase Style**	Ivoclar	10	9.0	385-515	V/B	1100
**Bluephase Style M8**	Ivoclar	10	9.0	430-490	B	800
**TransLux2Wave**	Kulzer	8	7.5	385-510	V/B	>1400 (Cure Rite)
**Scanwave Prototype**	Acteon	7.5 (Multifiber) 7.5 (Monofiber)	6.9 7.4	390-510	V/B	N/A
**S10**	3M	10	9.6	430-480	B	1200
**Deep Cure**	3M	10	9.6	430-480	B	1470
**Bluephase 16i**	Ivoclar	8 13	7.4 11.8	430-490	B	1600
**Supercharged Mini LED**	Acteon	7.5	7.0	420-480	B	2000
**SK-L036**	Spark	8	7.5	420-480	B	2200
**Cybird XD**	Dentazon	8	7.2	430-490	B	1500 & 2700
**S.P.E.C 3**	Coltene	8 11	7.4 10.0	430-490	B	1600 & 3000-3500
**BA Ultimate 1400**	BA International	8	7.0	400-480	V/B	1400
**Xlite 3**	3H Dental	8	6.5	385-515	V/B	1100
**BLAST lite**	First Medica	8	7.2	not available	B	2200
**Omega LED**	O’Ryan	8	7.6	not available	B	450
**Bluephase C8**	Ivoclar	8	7.4	420-480	B	800
**JAS-2001 B**	Online	8	6.6	400-490	B	>2700
**Demetron A2**	Kerr	4 8 11 13	3.7 7.4 9.9 12.5	450-470	B	1100
**Light curing unit**	**Manufacturer**	Active diameter** (mm)	**Wavelength Range (nm)**	**Violet/ Blue Chip**	Peak Irradiance* (mW/cm2)	
**Xlite 2**	3H Dental	7.8	380-515	V/B	1600	
**Smartlite Focus**	Dentsply	7.5	430-490	B	1000	
**Demi Ultra**	Kerr	7.9	438-485	B	1100-1330	
**SiriusMax**	ND	9.1	430-490	B	1200 & 3000	
**Pencure VL-7**	Morita	8.8	420-480	B	1000	
**Pencure VL-10**	Morita	8.8	420-480	B	1000	
**Fusion 3.0 Blue**	Dentlight	9.6	420-490	B	2700	
**Fusion 3.0 Violet**	Dentlight	9.6	390-430	V	2700	
**Fusion 5.0 Blue**	Dentlight	9.6	415-490	B	4000 (Plasma)	
**Fusion 5.0 Violet**	Dentlight	9.6	390-430	V	2700 (Plasma)	
**Valo**	Ultradent	9.6	395-480	V/B	1000	
**Valo Grand**	Ultradent	11.7	395-480	V/B	3200 (Xtra)	
**Radii Plus**	Ultradent	7.0	440-480	B	1500	
**Coltolux**	Coltene	7.8	450-470	B	NA	
**FLASH lite Magna**	Den-Mat	11.0	440-490	B	> 1100	
**i LED**	Woodpecker	7.8	420-480	B	2300 (P1)	
**DTE LUX-1**	Woodpecker	6.4	420-480	B	850-1000	
**Xlite 4**	3H Dental	7.0	385-515	V/B	2000	
**Radii Xpert**	SDI	7.5	440-480	B	1500	

* Manufacturers stated.

** Actual light emitting diameter and area. For Type II LCUs the external head diameter is neither relevant nor reported by the manufacturers. Irradiance and wavelength ranges are drawn from manufacturers’ literature. Tables adapted from Shortall et al. 2021 [27].

### High irradiance curing

**Figure 8:**
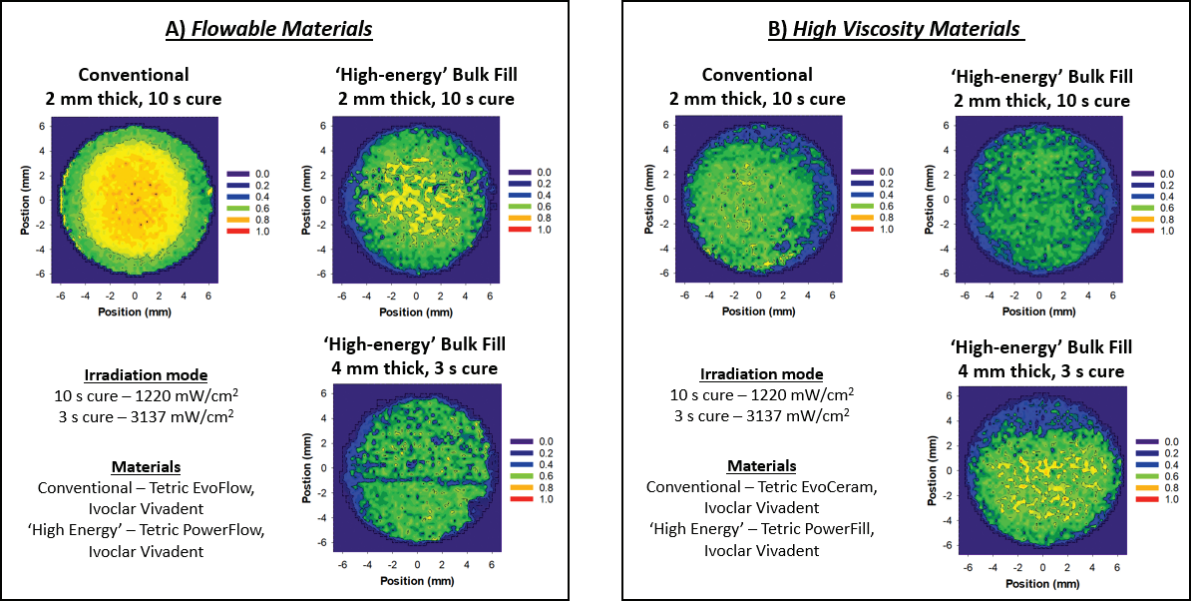
Optimisation of light-material interactions in some commercially available materials. The figure shows mapped degree of conversion measured after 24 hrs (cured using Powercure light curing unit, Ivoclar Vivadent) in 250 µm incremental step size (3,025 pixels per image). Spatial and temporal degree of conversion is dependent on material type (A – flowable materials, B – High viscosity materials), thickness and curing method. A significantly lower degree of conversion (~40%, p<0.05) can be seen around the periphery in all groups. However, the materials optimised for so called ‘high energy’ curing exhibit relatively homogenous cure profiles within the central 10 mm. The conventional materials exhibits significantly lower degree of conversion in the outer 4 mm of the specimens which was more pronounced in the flowable material (unpublished data).

**Figure 9:**
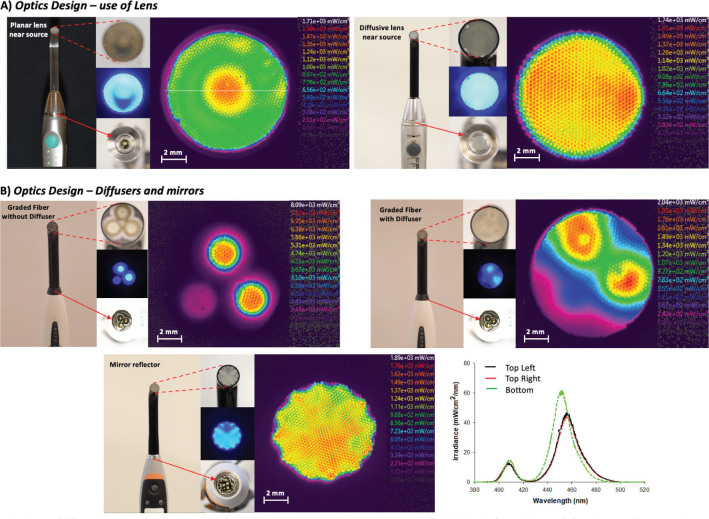
(A) Beam profile homogenisation in two modern Type I dental light-curing units. (A) the use of lenses near the LED source to improve far field emission patterns for collection into a graded fiber in the S10, 3M ESPE light (left) and the Deepcure, 3M ESPE light (right). On the left is a non-diffusive planar lens which results in Guassian beam profile that has a ‘hot-spot’ at the centre of a 10 mm graded fiber exit. On the right is a hemispherical diffusive lens which produces a much more homogenous beam profile. (B) The use of diffusers and mirrors for homogenisation of example multipeak light-curing units. The presence of multiple diodes (2 × visible, 1 × violet) produces an inhomogenous beam profile at the source with localised LED-wavelength dependent irradiance. The top two images show the same light source (Bluephase Style, Ivoclar Vivadent) which has a protective planar optical glass lens which is unlikely to affect the beam profile. The image on the top left is a graded fiber light guide without a diffuser which subsequently results in localised LED-wavelength dependent irradiance at the tip exit. The image on the top right is a graded fiber light guide with a diffuser which subsequently results in a more homogenous irradiance at the tip exit without affecting the spectral irradiance. The image on the bottom shows a similar light (Bluephase Powercure, Ivoclar Vivadent) with a reflective mirror housing near the four LED sources (3 × visible, 1 × violet) which produces the homogenous beam profile at the tip exit of a graded fiber without a diffuser.

In general, the trend and drive for LCU development has been the desire to reduce curing time by increasing the optical power under the presumption that material properties and optimal polymerisation are solely dependent on the energy delivered with a reciprocal relationship between exposure time and irradiance (power per unit area) [15, 94]. However, the validity of this relationship (i.e. exposure reciprocity) is multifactorial and depends on both material composition and how the light is delivered [15, 46]. Nevertheless, with lasers and laser modulation becoming more affordable, the most modern developments using diode lasers also exploit this trend and aim to further reduce curing time (1 s for 2.5 mm) through much higher power outputs (~1.4–2.0 W) and supposedly provide more consistent dispersion of energy and power at any distance (e.g. Monet, introduced in 2021) with the manufacturers claiming more reliable, more homogenous and complete cure through depth [29, 30, 34, 45, 95]. Indeed, these claims need to be confirmed and only likely to be valid under certain conditions due to limitations in materials chemistry and physics which suggest that the process of curing dental materials is a quantum process and strictly not dependent on the energy delivered to the surface [60, 94]. Despite this, modern lights that allegedly deliver high energy using high irradiances actually deliver less energy to materials in short 1–3 s exposures compared with 20 s exposures from LCUs delivering 1,000 mW/cm^2^ (a 20× increase in irradiance, that is 20,000 mW/cm^2^ would be needed to even match the energy delivered of the latter for a 1 s cure) [34]. Nonetheless, Rocha et al. [30] tested the depth of cure of several market leading conventional and bulk fill materials (A2 shade) using a laser diode and demonstrated a depth of cure of at least 1.5 mm in 1 s. This is likely due to optimised light-material interactions that improve light transmission and curing efficiency. On one hand, the material is optimised in terms of its filler-resin refractive index and filler loading which reduces interfacial scattering and attenuation, the photoinitiator chemistry which is more efficient and more reactive, the polymer chemistry to improve cure kinetics, and the pigment/dye concentration which reduces absorption to allow deeper transmission of light and more homogenous cure through depth with high irradiances at short exposure times and low energy (Figure 8) [9]. On the other hand, the optimisation of LCU technology (e.g. high power diodes and modern electronics) to deliver higher irradiances, and optimised light delivery systems (e.g. efficient fibre light guides, lenses, diffusers, reflectors, and mirrors) that produce more homogenous and collimated beam profiles also contribute to this effect (Figure 9). However, whether adequate polymerisation can be achieved with high irradiances and short exposure times despite these modern developments in all types of materials including low viscosity conventional flowable materials with different curing characteristics [46], darker shades with different attenuation properties [94, 96] or materials that contain other photoinitiators with inadequate absorption characteristics and high molar absorptivity [12, 15] is yet to be seen. To date there exists only a limited number of peer-reviewed scientific literature on laser diode curing of dental materials thus a limited evidence base that neither conclusively supports or contradicts the effectiveness of high irradiance and short exposure time curing using laser diodes for wider adoption.

### Beam profile

Given the fact that LCUs vary in many aspects (e.g. wavelength, type of light source, number of diodes, the relative positioning of the diodes, the optics used for light delivery and tip diameter), the beam profile is also a source of variation (which itself can be affected by methods used for beam profile recording (Figure 7D) [27, 40]. QTH-LCUs produce highly divergent light and spherical light distribution that is delivered through fibre bundled light guides which mitigates the heating effects from the main unit of the LCU but at the same time produces homogenous beam profiles at the tip. For LED and diode laser lights, the optical characteristics such as beam angle and beam profile are controlled by the design of optics. This includes the shape of the reflector, the size and design of the diode, the distance from the chip surface to the top of the housing of the lens system, and the geometry of the lens. LED emission profile can generally be divided into two classes, edge emitters and surface emitters. Most surface emitters such as the ones used in dentistry exhibit a Lambertian emission pattern where the profile (intensity) is proportional to the cosine of the emission angle. In contrast, edge emitters typically emit light that is not symmetrical from a small region (~50 µm in diameter). At the light emitting source, collimation can be achieved through reflective cup housings and far field emission patterns can be improved using a combination of various lenses (planar, hemispherical and parabolic). The light can then be transferred into a collection or a second projection lens system for delivery during light curing. In dentistry, there are two principal delivery methods [27]; either a Type I unit that uses a light guide (usually a graded optical fibre bundle) to collect the light, or a Type II unit which incorporates the LED chipset directly in the unit head for projection of the light. The latter is possible because LEDs and laser diodes produce minimal heating at the light source compared with QTH-LCUs. In the absence of any optical engineering to optimise optical characteristics, single diode LCUs transmitted through simple graded fibre light guides exhibit Gaussian beam profiles that mimic the beam profile of the LED source, which may be problematic for homogeneity of cure [35, 97–98]. Light guides of Type I LCUs also vary according to material, taper and entry and exit diameters which further affects power output and beam profile [27]. Type II light guides can vary according to materials used (e.g. plastic or glass) and the type of lens (e.g. profile curvature and shape) which can also cause variation in the beam profile. In addition, the presence of multiple diodes and multiple wavelengths without proper beam homogenisation, coupled with the fact that many commercial materials now contain a combination of initiators has led to differential surface cure and differential cure through depth, particularly with short exposure times [27, 31–35, 97, 98]. This necessarily necessitates the use of prolonged cure times to achieve homogenised cure [35]. Light curing unit manufacturers have also attempted to reduce such differential cure by sophisticated optical engineering of modern lights to improve beam homogenisation. This includes the use of diffusers, lenses (including diffusion type lenses that may contain embedded glass particles to scatter the light into larger angles), reflectors as well as ‘hybrid’ type methods that incorporate both Type I and Type II features (Figure 9) [99].

### Spectral output

**Figure 10:**
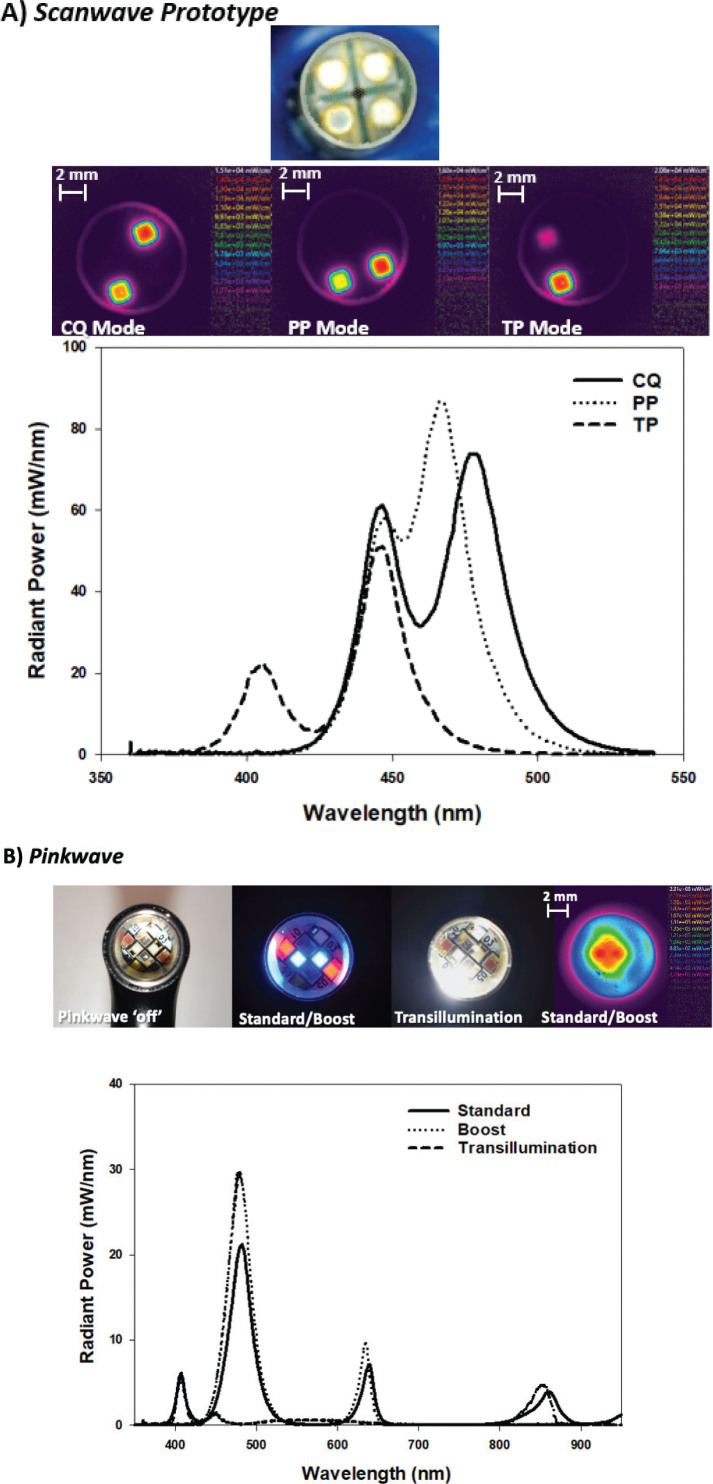
Images of the light guides, beam profiles and the spectral radiant power of multiwavelength light curing unit (LCU) technologies. (A) the Scanwave Prototype by MiniLED^TM^ with patented wavelength scanning technology that allows selection of the most appropriate spectral output mode and radiation time for any possible material and clinical situation. The sequential activation of different diode wavelength combinations allows delivery of a broad spectral output albeit with localised hotspots in beam profile. (B) the Pinkwave ^TM^ LCU (Vista Apex), equipped with patented ‘Quadwave^TM^, technology (UV – 405 nm, blue – 480 nm, red – 640 nm and NIR – 860 nm) to deliver a pink light to supposedly enhance cure. It is important to note, neither light has been optimised for beam profile.

The light produced by LCUs can also vary in spectral output (including its peak wavelength(s), the full width half maximum which describes the wavelength distribution and shape) (Figure 7B). Quartz tungsten halogen lamps necessitates the use of bandpass filter to block unwanted radiation including UV (<400 nm) and green, red and IR (>~550 nm) to improve thermal and photobiological safety [4, 7, 58, 60]. However, LEDs are relatively narrow band with typical spectral emission between ~440 and 480 nm and laser diodes are monochromatic (peak wavelength ~450 nm) centred around the peak absorption of CQ (~470 nm) [35]. While the former allows for effective spectral overlap with the absorption profiles of most dental photoinitiators (e.g. CQ, acylphosphine oxides and Ivocerin), the latter technologies are not effective at initiating polymerisation in materials with shorter wavelength initiators unless multiple diodes, often with different wavelengths are used. Modern trends in LED-LCU manufacturing therefore include ‘broad spectrum’ units which incorporate LEDs in the visible-violet (~390–405 nm), blue (~450–480 nm) as well as intermediate ranges of violet-blue wavelengths (~410–450 nm) (Figure 7B and Table 1) [27]. To this end, innovative technologies that allow multiple wavelength delivery have also appeared within the dental LCU market which offer scanning technology that allows dentists to select the most appropriate spectral output mode and radiation time for any material and clinical situation (Figure 10A). The sequential activation of different diode wavelength combinations throughout the irradiation cycle in ‘full scan’ mode allows the four different diodes to deliver a broad spectral output between 390 and 510 nm, supposedly allowing cure of all photocurable dental polymer-based materials, irrespective of photoinitiator chemistry with reportedly good conversion at depth while minimising heating effects [38]. Another recently marketed light is equipped with so called ‘Quadwave’, technology, which uses four different wavelengths (UV – 405 nm, blue – 480 nm, red – 640 nm and NIR – 860 nm) to deliver a pink light that supposedly enhances curing performance (Figure 10B) [34, 100]. The manufacturers claim that increased polymerisation is achieved by the NIR wavelengths, presumably through the increased heating as reported in a pulpal temperature rise *in vitro* study when low and high viscosity bulk fill composites were cured for 20 s compared with a laser diode and other contemporary LED LCUs used in standard modes of ≤10 s [101]. It is likely that the increased heat from the IR wavelengths extends the diffusion period of propagating molecules and therefore increases the extent of polymerisation [102, 103] although this is yet to be confirmed. The same light also features the first curing device as far as the authors are aware that has a built in transilluminator (although other lights have been developed which require attachments for this purpose) that delivers a bright white light which the manufacturer further claims can help diagnose conditions such as cracks, interproximal caries or to identify canal orifices. Indeed, the UV component of LED LCUs has also been promoted for caries, cracks and oral soft tissue lesion diagnosis as well as red wavelengths delivered by a red-light module head in dental LCUs promoted for PDT applications in combination with a photosensitiser for disinfecting root canals; these are emerging areas of shining lights in dentistry.

## Photonic energy for diagnostics and therapeutics

### Transillumination and fluorescence

The detection of dental caries has classically relied on a visual-tactile inspection using an explorer on the suspected lesion, supported by radiographic examination. While these methods have been reliable for the detection of caries, confirmation by radiograph requires 30–40% teeth demineralisation before lesions are detectable [104]. Radiographs also require the use of ionising radiation which is a major concern in healthcare [105]. Accurate early diagnosis is crucial for appropriate care management of caries by preventative or interceptive therapy. Several technologies have been introduced for early caries detection and include electric conductance measurements, ultrasound, and bioluminescences (e.g. fluorescence and transillumination), and despite fluorescence and transillumination showing promising results [106], methods to assist visual and radiographic means of caries detection and caries diagnosis remains a challenge for the dental profession.

Quantitative laser fluorescence relies on the projection of visible light (ʎ = 600–700 nm, typically from a laser diode) onto suspected caries lesions which is then absorbed by endogenous fluorophores [107, 108]. More recently, a caries detecting oral rinse (LumiCare^TM^) has been developed which contain proprietary fluorescent starch nanoparticles that allow active initial caries lesions to be detected using a dental curing lamp [109]. Similar to dental photoinitiators, absorption by the endogenous fluorophores or the fluorescent starch nanoparticles leads to excitation of electrons, thermalisation of energy and eventually a return to ground state with the emission of longer wavelength (typically NIR), lower energy radiation. Consequently, the carious lesion appears dark and can be distinguished from normal, healthy tissues [107, 108]. However, due to tissue characteristics of enamel, there is poor sensitivity for the detection of enamel lesions and depth resolved images of lesions severity or demineralisation are not possible with direct fluorescence techniques [107]. Transillumination is the use of high irradiance visible light to help define normal from abnormal structures or functions, the principal of which is based on the diffusion of light in tissues which have different densities and composition. The optical properties of mineralised teeth appear different to demineralised areas which present with more pores and interprismatic water that leads to more scattering which results in demineralised lesions appearing darker [110]. Due to the differences in demineralisation and water content, shallow and mild demineralisation appear lower in contrast and deeper lesions appear greater in contrast [104]. Fiber optic transillumination (FOTI) is a fairly modern development of a classic diagnostic aid advocated some 30 years ago that never gained wide acceptance possibly because of the failure to appreciate that the technique, like any other, requires an extended learning phase [111]. FOTI and a digital version known as DFOTI both use short wavelength visible light which increases scattering in enamel which reduces contrast and therefore its sensitivity to detect lesions. Near-infrared light transillumination, patented in 2006, has subsequently gained popularity due to improved contrast and sensitivity [112]. The principle remains the same but utilises a wavelength range of 780–1,600 nm which improves transmission through gingival tissue and bone to be effectively scattered by tooth tissues. The reflected light is then captured by a charge coupled device (CCD) sensor and converted into an NIR image that can be used for diagnostics.

### Therapeutic applications of light

#### Photobiomodulation

There is a myriad of applications of lights in dentistry which harness its photonic energy, one application is its use for therapeutic purposes. PBM is the direct application of light that is usually delivered via a low power (≤500 mW) laser or LED to stimulate (or inhibit) cell responses to elicit physiological changes that promote tissue healing, reduce inflammation and induce analgesia. PBM uses lasers or LEDs which typically deliver pulsed or continuous wave light in the 600–1,000 nm spectral range (red to near infrared) for 30–60 s exposures per treatment, with irradiances of 5 mW/cm^2^ to 5 W/cm^2^, generated by devices with powers of 1 mW to 10 W [113]. Recent studies have also demonstrated the ability of shorter wavelength light (405 nm) to stimulate positive PBM responses in cells and tissues including differentiation and mineralisation of mesenchymal stem cells and odontogenic differentiation of dental pulp cells [114, 115]. Indeed, treatment outcomes are dependent upon a number of factors including target tissue type, tissue properties, and importantly wavelength and dosimetry [113–117]. Generally, the poor acceptance of PBM has been linked to a poor understanding and reporting of photophysical and radiometric properties, as well as the lack of a full elucidation of the biological processes of PBM [117]. If the incorrect irradiation parameters (including wavelength, exposure time, irradiance and dose) are used, then treatment will likely be ineffective and is demonstrated by the existence of a biphasic dose-response curve known as the Arndt Schulz curve [113–117]. However, while PBM treatments are reportedly dose-dependent, the existence of a reciprocal relationship between exposure time and irradiance is unlikely and like photocuring of dental materials, the process is likely to be a quantum process that does not depend on the energy delivered but rather independently dependent on the combination of irradiance and exposure time used [118].

Unlike other laser treatments, PBM is not an ablating or heating based therapy but a photophysical and photochemical response at various biological scales. Similar to dental photoinitiators, the mechanism involves the absorption of light by a photosensitiser which in this case is endogenous to cells rather than exogenous as in the case of similar treatments such as PDT [119, 120]. While the precise mechanism of the former is yet to be fully understood, the most accepted theory suggests chromophores within the mitochondria absorb light causing a cascade of events that release bound nitric oxide, allow the rebinding of oxygen to restore cellular respiration, increase ATP and reactive oxygen species (ROS) production and eventually lead to gene transcription and positive therapeutic outcomes [113]. Other plausible explanations for PBM have also been proposed, which include action through photoreceptors and extracellular signalling molecules [121, 122] but have neither been proven or disproven although it is likely that a combination of mechanisms are involved in positive therapeutic outcomes.

In comparison to other biomedical applications of PBM which are supported by more than 300 randomised double-blind placebo controlled clinical trials and expert consensus reports [123–130], dental applications are less well documented. However, the evidence base behind its usefulness in dentistry is rapidly increasing [114–118, 122, 131–133]. There now exists encouraging data for PBM applications in a wide range of oral hard and soft tissues which cover a number of key dental specialities, including endodontics, periodontics, orthodontics, paediatric, prosthodontics and maxillofacial surgery [131]. Promising applications in dentistry include its use after surgical placement of titanium implants to improve implant stability, attachment and osseointegration [134]. A recent review also highlighted the positive influence of PBM on the outcome of regenerative endodontic procedures [133]. Recent data further supports the use of PBM for various oral pathology applications including its use as an adjunctive therapy for oral mucositis [135]. To this end, the National Institute for Health and Care Excellence (NICE, UK) has approved and recommended PBM for preventing or treating oral mucositis caused by radiotherapy or chemotherapy which represents a milestone in dentistry for its acceptance and wider application [136].

### Photodynamic therapy and photodisinfection

In contrast to PBM, the mechanism of PDT is better understood and utilises light indirectly to trigger an exogenous photosensitiser to produce ROS (e.g. hydroxyl radicals, superoxide anions and singlet oxygen species) that destroy infecting molecules that cause disease. There are a number of clinically relevant photosensitisers for dentistry which have been indicated for different applications, the most common being 5-aminolevulinic acid (ALA) [137]. Current data indicates that PDT is an effective adjunctive tool for treating oral disease in several dental specialities including oral and maxillofacial surgery, oral medicine and oral surgery for the treatment of pre-malignant and malignant lesions of the head and neck region, including the oral cavity [137]. This is because photosensitisers such as ALA show a selective affinity for tumours of vascular tissue cells including precancerous and cancerous lesions in the oral cavity. Photodynamic therapy has also recently been used for the diagnosis of lesions in the oral cavity which represents an important advancement in dentistry, again achieved through selective accumulation of the photosensitiser in lesions which leads to increased fluorescence compared with healthy tissues [137]. Other uses in oral surgery include prevention and treatment of alveolar osteitis and post-extraction pain [137]. Photodynamic therapy has further also been used in other specialities, including endodontics (photodisinfection of the root canal) [137], periodontitis (photodisinfection of periodontal pockets), and implantology (treatment of peri-implantitis) [119, 120]. Many PDT studies have also examined the effect of light on the lactic acid producing bacteria, *Streptococcus mutans* using photosensitisers such as toluidine blue (ʎmax- 630 nm), methylene blue (ʎmax- 660 nm), curcumin (ʎmax- 450 nm) and disulfonate phthalocyanine (ʎmax- 600–700 nm) [138, 139]. While the technique has shown to reduce bacterial and fungal contamination *in vivo* [140], and when used in dentine, reduced *Streptococcus mutans* counts in deep carious lesions [141], it’s limited clinical success in this area of dentistry is likely due to problems associated with the diffusion of the photosensitiser within biofilms [142]. To overcome this, recently, the direct application of light, similar to PBM albeit at shorter wavelengths (typically <450 nm) and higher energy, has been advocated for direct bactericidal effects [116, 143]. In this case, locally derived chromophores in bacteria known as porphyrins which are a group of heterocyclic organic compounds that are essential for bacterial synthesis of heme, are complexed to proteins and possess a Soret band that absorb blue light (400–420 nm). Whist these porphyrins are the main chromophores that absorb blue light, flavins and flavoenzymes also represent potential chromophores for direct blue light photodisinfection [144]. Absorption of light results in the excitation of electrons to a high energy state and oxidation of molecules containing such chromophores [115, 116]. This subsequently results in the release of ROS that are capable of exerting antibacterial effects through oxidative cell membrane and DNA damage. A recent study targeted protoporhyrin IX, an intracellular pigment of *Porphyromonas gingivalis* without an external photosensitiser to mitigate risks of cytotoxicity in stained tissues during PDT treatments and demonstrated the ability to achieve direct photodisinfection [143]. Another recent study demonstrated effective inhibition of cariogenic bacteria in biofilms using wavelengths centred at 405 nm as well as effective decontamination of dental tissues [145]. Similar to PBM, these effects are dose dependent and rely on specific irradiation parameters (irradiance and exposure time) for effective bactericidal effects with reduction in bacterial viability being directly related to dose [116, 143, 145].

### Light-tissue interactions

The use of light in biomedical applications whether it is for biomaterials processing and fabrication of restorations, or to obtain therapeutic effects generally using PBM, PDT and photodisinfection is complicated due to specific dosimetry requirements in terms of irradiance, exposure time and energy delivered. The presence of biological tissues and materials which scatter, absorb and attenuate light needs careful consideration for treatment planning and effective delivery of light of sufficient irradiance, duration and energy for any given application. Biological tissues differ in both two and three-dimensional architecture morphologically, molecularly, physically, and biologically between different species, donors of the same species, anatomical location of the tissue, within the same location (tissue heterogeneity) and other sites, and between other specific characteristics such as colour/shade and age of tissue amongst many other factors [146]. Secondary medical care and other environmental effects can also influence many of these factors and also need to be considered during treatment planning for successful treatment outcomes [146]. Such variability leads to differences in optical properties that include absorption, reflection, refraction, scattering in tissues, scattering at the interfaces of tissue components and transmission through tissues. Hard tissues have physiologically and anatomically complex structures, are non-vascularised, mineralised, and exhibit heterogeneity in terms of micro and macroscopic structure. Dentine in particular has heterogeneously orientated microscopic channels called dentine tubules (~1–4 µm diameter) that run between the dentinoenamel junction in the crown and the dentinocemental junction in the root to the inner wall of the pulp chamber at ~90 degree angles. Within this microscopic channel system, there also exists branching nano-canalicular systems that connects microtubules to each other. These nano-canals have a size range from 300 to 1,000 nm with major branches at the terminal ends of the microtubules and fine branches every 1–2 µm diverging at 45 degree angles [147]. Each tubule is surrounded by a matrix of needle shaped, hydroxyapatite like crystals, in a protein matrix which is largely composed of collagen [148, 149]. However, tubule shapes, orientation, size and density vary between different regions of dentine and these microscopic regional differences can lead optical differences in what is supposedly the same tissue. For example, from the outer surface of dentine to the area nearest to the pulp in specimens from the buccal region have tubules that follow an S-shaped path [150]. In contrast, specimens from the occlusal surface have a more linear path and specimens from the oblique region will have a non-linear but less sigmoidal shaped path than specimens from the buccal region and occlusal regions. In addition, the diameter and tubule density is generally greater near the pulp compared to the outer surfaces with tubules tapering out towards the surface [150]. The importance of considering light-tissue interactions was highlighted in a recent study which explored the interaction of light with dentine to optimise light delivery for photodisinfection of dental tissues [116]. The study reported that light transmission is dependent on a number of tissue-related factors, including the thickness of the dentine, its microstructure (i.e. the shape and direction of the tubules with respect to the direction of the light delivery) as well as the dentinal tubule density. Optimised light transmission was reported through occlusal and oblique dentine in comparison to buccal dentine which resulted in effective decontamination of dental tissues [116]. The same light-tissue interactions are likely to govern PBM outcomes of dental pulp cells which has been reported in *in vitro* conditions and *ex-vivo* transdentinal models at specific radiant exposure [151, 152], although with different wavelengths compared with photodisinfection. Indeed, transmission of light through dentine is also wavelength dependent and a mean power loss of approximately 40% in 0.2 mm thick specimens has been reported with near infrared light exhibiting higher transmission than blue and red light [153, 154]. Firstly, the scattering coefficients are dependent on wavelength [149]. Secondly. the chemical composition of dentine differs between regions due to compositional variation in minerals such as hydroxyapatite [155], as well as the presence of natural chromophores and pigments such as pyroles and porhyrins [155]. Short wavelengths are absorbed by proteins such as albumin, transferrin, tenascin and proteoglycan found in tissues and in remnant dentinal fluids [149, 156]. Hydroxyapatite has relatively strong absorption at wavelengths <600 nm [155] but exhibits reduced absorbance between ~600 and ~1,300 nm. Increasing transmission with increasing wavelength (400–850 nm) has indeed been previously reported by Turrioni et al. [154]. Meglinski et al. [157] also demonstrated that even in the same tissue, distinct wavelengths gave rise to different absorption coefficients. Thus, the effectiveness of any light-based treatment or diagnostic procedures that involves delivering light through biological tissues depend on the wavelength of light, the anatomical location, the scattering coefficient and the absorption coefficient. While recent studies have attempted to understand these effects for photodisinfection and PBM of dental tissues, the same principles also apply for transillumination for diagnostic purposes and trans-dentinal light curing of photocurable restorative materials. Further work is thus needed in these areas to optimise these light-tissue interactions.

## Conclusions

Photonic energy has benefited dentistry by providing unique advantages for a range of applications from controllable and effective curing of dental materials to transillumination for diagnostics, and therapeutic applications including PDT, PBM and photodisinfection. However, successful clinical treatment outcomes critically depend on the delivered photon energy and how it is delivered in terms of irradiance and exposure time. These factors are governed by light-material interactions and light tissue-interactions, which often limit the ability to deliver sufficient irradiance and energy through depth due to reduced light transmission.

The application of photonic energy in dentistry is likely to increase with improvements in materials chemistry, light delivery technology, and improved understanding towards the interaction of light with materials and biological tissues that may improve irradiance through depth and at therapeutic target sites, respectively. For the latter, the increasing evidence base for the diagnostic and therapeutic application of light in dentistry is likely to lead to greater adoption of light-based technologies in a range of dental specialities and for a range of dental treatments.

## Data Availability

The unpublished data presented in this review is available on request.

## Key messages

The photocuring of dental materials is limited by inefficient light transmission and limitations in materials chemistry.

New developments in photoinitiators and dental lights aim to deliver more homogenous cure through depth as well as a reduction in cure time.

The increasing evidence base for the diagnostic and therapeutic application of light in dentistry is likely to lead to greater adoption of light-based technologies in a range of dental specialities and for a range of dental treatments.
